# Clonal chromosomal and genomic instability during human multipotent mesenchymal stromal cells long-term culture

**DOI:** 10.1371/journal.pone.0192445

**Published:** 2018-02-12

**Authors:** Victoria Nikitina, Tatiana Astrelina, Vladimir Nugis, Aleksandr Ostashkin, Tatiana Karaseva, Ekaterina Dobrovolskaya, Dariya Usupzhanova, Yulia Suchkova, Elena Lomonosova, Sergey Rodin, Vitaliy Brunchukov, Stanislav Lauk-Dubitskiy, Valentin Brumberg, Anastasia Machova, Irina Kobzeva, Andrey Bushmanov, Aleksandr Samoilov

**Affiliations:** 1 State Research Center, Burnasyan Federal Medical Biophysical Center of Federal Medical Biological Agency of Russia, Moscow, Russia; 2 Department of Medical Biochemistry and Biophysics Karolinska Institutet, Stockholm, Sweden; Virginia Tech, UNITED STATES

## Abstract

**Background aims:**

Spontaneous mutagenesis often leads to appearance of genetic changes in cells. Although human multipotent mesenchymal stromal cells (hMSC) are considered as genetically stable, there is a risk of genomic and structural chromosome instability and, therefore, side effects of cell therapy associated with long-term effects. In this study, the karyotype, genetic variability and clone formation analyses have been carried out in the long-term culture MSC from human gingival mucosa.

**Methods:**

The immunophenotype of MSC has been examined using flow cytofluorometry and short tandem repeat (STR) analysis has been carried out for authentication. The karyotype has been examined using GTG staining and mFISH, while the assessment of the aneuploidy 8 frequency has been performed using centromere specific chromosome FISH probes in interphase cells.

**Results:**

The immunophenotype and STR loci combination did not change during the process of cultivation. From passage 23 the proliferative activity of cultured MSCs was significantly reduced. From passage 12 of cultivation, clones of cells with stable chromosome aberrations have been identified and the biggest of these (12%) are tetrasomy of chromosome 8. The random genetic and structural chromosomal aberrations and the spontaneous level of chromosomal aberrations in the hMSC long-term cultures were also described.

**Conclusions:**

The spectrum of spontaneous chromosomal aberrations in MSC long-term cultivation has been described. Clonal chromosomal aberrations have been identified. A clone of cells with tetrasomy 8 has been detected in passage 12 and has reached the maximum size by passage 18 before and decreased along with the reduction of proliferative activity of cell line by passage 26. At later passages, the MSC line exhibited a set of cells with structural variants of the karyotype with a preponderance of normal diploid cells. The results of our study strongly suggest a need for rigorous genetic analyses of the clone formation in cultured MSCs before use in medicine.

## Introduction

Quality control and standardization of cellular biomedical products are important for favourable outcomes of cell therapies. Even with optimal conditions of cultivation *in vitro*, there can be a change of properties and loss of functional characteristics of the tissue *in vivo*. This can be attributed to a lack of regulatory humoral factors, loss of intracellular and intertissue interactions, genetic variability and spontaneous mutagenesis. Mutation variability is a genetic variation among members of the same species, which leads to the emergence of genotypic and phenotypic differences from parent forms. Genetically modified cells that may occur as a result of adaptation to culture conditions give rise to new cell clones. If a chromosome aberration increases the rate of proliferation or leads to other changes, which are useful for cells, then the faster growing cells could rapidly displace the slower cells with normal karyotype. In the cytogenetics of continuous cell lines, this process is called "karyotypic evolution" and is subdivided into two stages, namely establishment and stabilization. The stages are different in karyotypic variability of the cell populations and in peculiarities of clone selection. “Establishment” is characterized by genetic heterogeneity of the cell population and selection of cell clones that are best adapted to the existence in vitro and may last for a prolonged period of time before emergence of a stable population. During “stabilization” the cell population of lines usually consists of one predominant cell clone and some minor subclones [1, 2].

Development of cellular biomedical products must preserve the original or intentionally modified properties of the cells. This includes prevention of formation and evolutional selection of clones. In majority of clinical trials involving MSCs published to date, the cells are used at early passages (no more than passage 5). However, cells with clonal aneuploidy and translocations have been described already at 1^st^ and 4^th^ passages of various MSC lines [3,4,5]. An extensive body of data suggests that MSCs are relatively genetically stable during culturing in vitro [6–9, 10, 11]. Nevertheless, there are evidences of increase in the rate of mutagenesis in MSCs with prolongation of the culture period [3–5, 12–14]. It is necessary to consider separately abnormal cell clones in MSC lines, obtained from donors with constitutional karyotype abnormalities, such as balanced chromosomal translocations (ex. Robertsonian Translocations) or sex chromosomal mosaicism [3,4]. Rigorous testing of cellular biomedical products containing MSCs at various stages of development can prevent using genetically abnormal cells in treatment of patients and, therefore, prevent possible complications of the cell therapies [15,16]. Studying genetic variability and clone formation can help provide insights into the mechanisms of MSCs transformation and facilitate the development of effective strategies for such testing. In this study, we have analysed chromosome and genome variability as well as clone formation during human MSC long-term culture.

## Methods

### MSC cultivation

Cell lines derived from the gingival mucosa of a healthy patient was taken from the collection of the Cryobank of the Center for Biomedical Technologies Burnasyan Federal Medical Biophysical Center of Federal Medical Biological Agency of Russia, Moscow. Samples were obtained with informed consent and in accordance with the Ethics Committee (Reference number: 5A/12.02.2016). The MSC cell line has been confirmed to all International Society for Cellular Therapy criteria, and had normal male karyotype 46, XY. Cryopreserved cells were placed in a water bath at 37°C for defrosting and the cultivation was started from passage 5. Cell suspension was placed in culture flasks (surface area 75 cm^2^) with concentration of 0.6–0.8x10^6^ per flask, in low glucose Dulbeco’s Modified Eagle Medium (DMEM) supplemented with 10% fetal calf serum (Stem Cell, USA), L-glutamine and antibiotics (penicillin, streptomycin). Culturing was carried out at 37°C at absolute humidity and 5% CO_2_, until passage 26 (for approximately 6 month).

### Short tandem repeat analysis

For MSC authentication, the analysis of short tandem repeats (STR) was carried out using the "COrDIS Plus" kit for DNA identification of 19 STR markers and a human amelogenin locus. PCR-compatible lysis reagent "COrDIS Sprint" was used for DNA isolation (Gordis, Russia). The analysis included identification of 20 loci, including 13 classical systems of the combined DNA index—CODIS (D3S1358, D5S818, D7S820, D8S1179, D13S317, D16S539, D18S51, D21S11, CSF1PO, FGA, TH01, TPOX, VWA), 5 –ENFCI (D1S1656, D2S441, D10S1248, D12S391, D22S1045), and also SE33 and amelogenin. The evaluation was carried out three times.

### Evaluation of proliferation rate and immunophenotype

The dynamics of cell population growth was evaluated by cell increment multiplicity (number of cells obtained after current passage/number of cells inoculated at the previous passage). Surface markers were evaluated using a panel of monoclonal antibodies to CD45-FITC, CD73-PE, CD90-PE, CD105-APC, CD44-FITC, CD34-FITC, CD54-FITC, CD31-PE, CD133-PE, CD63-FITC, CD13-FITC, CD117-APC, HLA-DR-APC (BDBiosciences, USA). The percentage of cells expressing a certain antigen in MSC culture was calculated using flow cytometry (BD FACSCanto II).

### Fixation of MSC and cytogenetic sample preparation

A part of the cells (0.6×10^6^) was placed in the separate culture flask (75 cm^2^) and cultured for 1–2 additional days until 70% confluence was reached. Colchicine in a concentration of 0.04 μg/ml was added in the culture flasks and the cells were incubated for 4–6 h after which the cells were exposed to hypotonic solution (0.075 M KCL) for 9 minutes and fixed in methanol:glacial acetic acid (3:1) mixture.

### Cytogenetic studies

Cytogenetic preparations for karyotyping were prepared by G-banding and 24-colour staining of chromosomes ("24-Cyte", MetaSystems, Germany). Karyotyping was carried out at passages 6, 9, 12, 16, 18, 20 and 22. The calculation included metaphase plates with at least 45 chromosomes. To confirm the presence of an aneuploid cells clones, interphase FISH analysis was carried out using centromere-specific DNA probes (CEP6 (D6Z1), orange, CEP8 (D8Z1), green, Vysis, Abbott, USA). Denaturation, hybridization and washout were carried out according to the manufacturer’s instructions and contrasted with 4',6-diamidino-2-phenylindole (DAPI). Signal detection and subsequent metaphase analysis was done using the Metafer system and Metasytems’ ISIS software (Carl Zeiss, Metasystems, GmbH, Germany). Chromosome aberrations were described according to the International System of Human Cytogenetic Nomenclature (ISCN 2013) [17]. For the assessment of polyploidy, about 2000 routinely stained metaphase spreads were analyzed.

## Results

### Proliferation rate and MSC immunophenotype

Between passages 5 and 16, number of harvested cells at the end of each passage was approximately 4 times (4.2±1.9) higher than number of seeded MSCs at the beginning of the passage. A slight increase in growth rate was observed at passages 17–20, but at passages 23–26 the cells ceased to proliferate showing no increase in numbers at the end of the passages. Analysis of immunophenotype showed that during the whole time of cultivation up to passage 26, the cell line could be referred to as MSC in accordance with the requirements of International Society for Cellular Therapy [18]. High expression of CD73, CD90, CD105 and CD13 (99.7, 99.6, of 99.6 and 99.8%, respectively) and also low expression of markers of hematopoietic series (CD45, CD133, CD34, CD117, HLA-DR) with a tendency to the further reduction during cultivation, were detected.

### Short tandem repeat analysis and karyotype of MSC

**Fig 1:**
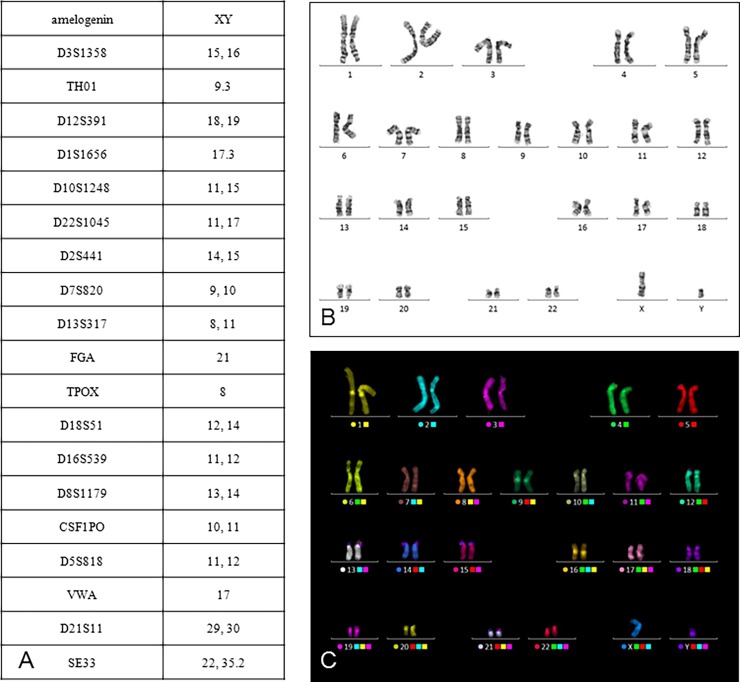
STR loci (A) and karyotype of the MSC at 6^th^ passage of cultivation: GTG (B) and mFISH (C) staining.

Analysis of short tandem repeats profiling was carried out on different passages to authenticate the cell cultures. The combination of STR loci and constitutional karyotype (Fig 1) corresponded to MSC data from the database of the Cryobank.

### Chromosome structure instability

Cells with chromosomal and chromatid fragments, deletions and translocations were taken into account. Overall, the karyotype of 426 cells at different passages were analysed (Table 1). Among them, 406 were analysed using mFISH and 20 using G-banding (at passage 6). Clonal aberrations were considered as single events, as their formation took place once and they were inherited in the clonal progeny. The total count did not include aberrations occurring in clonal cells. For instance, the additional translocation t(6;21)(p24;q22) in a clonal cell—46,XY,t(2;4)(p24;q12),t(6;12)(q24;q12) or polyploidy in clone 46,XY,del(6)(q21),t(6;14)(?;q32) (Tables 1 and 2) were not taken into account.

**Table 1:** Clonal and non-clonal chromosome aberrations and genome variations at different MSC passages.

Total passages	Total cells	Karyotype		
**1. GTG**		Normal cells	Cells with aneuploidy/ polyploidy	Cells with structural chromosome aberrations
6th	20	46,XY[18]	45,X,-Y 45,XY,-8	-
**2. mFISH**				
6th	32	46,XY[25]	45,XY,-11 45,XY,-12 45,XY,-17 45,XY,-22	46,XY,t(X;2)(p21;q14) 46,XY,t(2;7)(p23;p21) 46,XY,t(2;7)(q14;p15)
9th	40	46,XY[31]	45,XY,-2 47,XY,+6 47,XY,+8 45,XY,-17 45,XY,-21/ 92,XXYY	46,XY,chrb(1)(p35) 46,XY,chrb(5) 45,XY,-10,del(21)(q22)
12th	86	46,XY[73]	45,XY,-1 45,XY,-8 *45*,*XY-9[3]/* 91,XXYY,-9	45,X,chrb(X)(q25)Y,-8 **46,XY,t(2;4)(p24;q12),t(6;12)(q24;q12)**^**2**^ 46,XY,t(2;11)(q12;q14) 45,XY,-3,chtb(7)(p13) 46,XY,t(4;9)(p12;q32) 46,XY,t(10;17)(q22;q21) 46,XY,t(10;21)(q26;q22)
16th	87	46,XY[59]	*45*,*X*,*-Y[2]* **48,XY,+8,+8[7]**^**1**^ *45*,*XY[–9]* 45,XY,-11 45,XY,-14/ 92,XXYY	*45*,*X*,*-Y*,*t(9;12;18)(q13;p13;p11*.*2)* 46,XY,t(1;8)(q24;p23) 46,XY,t(1;20)(p13;q13) 46,XY,t(1;20)(p32;p12) 46,XY,t(2;6)(q37;p13),t(13;22;16)(q13;q12;q24) 46,XY,t(5;9)(p14;q34) 46,XY,t(5;21;8)(p10;q21;p21) 46,XY,chrb(6)(q22) 45,XY,del(7)(q31),-20 **48,XY,+8,+8,**chrb(22)(?)^**1**^ *45*,*XY*,*-9*,*chtb(20)(q12)* 46,XY,**t(9;13)(q13;q12)**^**4**^ 45,XY,-10,chrb(11)(p11) 46,XY,t(13;18)(q12;q12) 46,XY,t(13;22)(p11;q13)
18th	60	46,XY[40]	45,X,-Y 45,XY,-3 45,XY,-4 **48,XY,+8,+8[9]**^**1**^ 45,XY,-12/ 92,XXYY	46,XY,chtb(2)(q35) 46,XY,**t(2;4)(p24;q12),t(6;12)(q24;q12)**^**2**^ 45,XY,-6,chtb(5) 46,XY,t(8;14)(?;q32) 46,XY,**t(9;13)(q13;q12)**^**4**^ 46,XY,del(11)(p12;q23)
20th	61	46,XY[41]	*45*,*X*,*-Y[5]* 45,XY,-7 **48,XY,+8,+8[2]**^**1**^ 45,XY,-13 45,XY,-22	46,XY,t(1;5)(p36.3;p12) 46,XY,t(1;15)(q21;q22) 46,XY,**t(2;4)(p24;q12),t(6;12)(q24;q12)**,t(6;21)(p24;q22)^**2**^ 45,XY,t(3;16)(q28;q11),t(9;12)(p13;q13),-12 46,XY,**del(6)(q21),t(6;14)(?;q32)**,del(12)(q13)^**3**^ 91,XXYY,**del(6)(q21),**-**del(6)(q21),t(6;14)(?;q32)x2**^3^* 46,XY,t(6;21)(q11;p11.2) 46,XY,t(6;22)(?;q13) 46,XY,del(7)(q31) 46,XY,del(14)(q24)
22th	40	46,XY[27]	45,XY,-1 45,XY,-4 45,XY,-10 45,XY,-12 47,XY,+19/ 90,XXYY,-8,-8 92,XXYY[2]	46,XY,chtb(5)(q14) 46,XY,chrb(5)(?) 46,XY,**del(6)(q21),t(6;14)(q?;q32)**^**3**^ 46,XY,t(8;17)(q23;q21),-12 46,XY,t(14;20)(q31;?)

The number of cells is given in square brackets

^**1**^ –clone 1

^**2**^ –clone 2

^**3**^ –clone 3

^**4**^ –clone 4

*—clonal chromosome aberration in the polyploid cells.

**Table 2:** Frequency of chromosomal aberrations and breaks in MSC.

Passage	Aberrant cells	Breaks	Total cells	Fisher’s exact test	Percent of aberrant cells	Breaks for 1 cell
6	3	6	41	-	9.4	0.19
9	3	6	40	p≥0.05	7.5	0.15
12	7	15	86	p≥0.05	8.1	0.17
16	14	32	87	p = 0.032786	16.1	0.37
18	4	6	60	p = 0.004137	6.7	0.1
20	8	21	61	p = 0.010482	13.1	0.34
22	4	7	40	p≥0.05	10	0.18
total	40	87	415		10.13±1.27	0.21±0.04

Significant differences were found by comparing the number of breaks between passages: 12 and 16, 16 and 18, 18 and 20.

**Fig 2:**
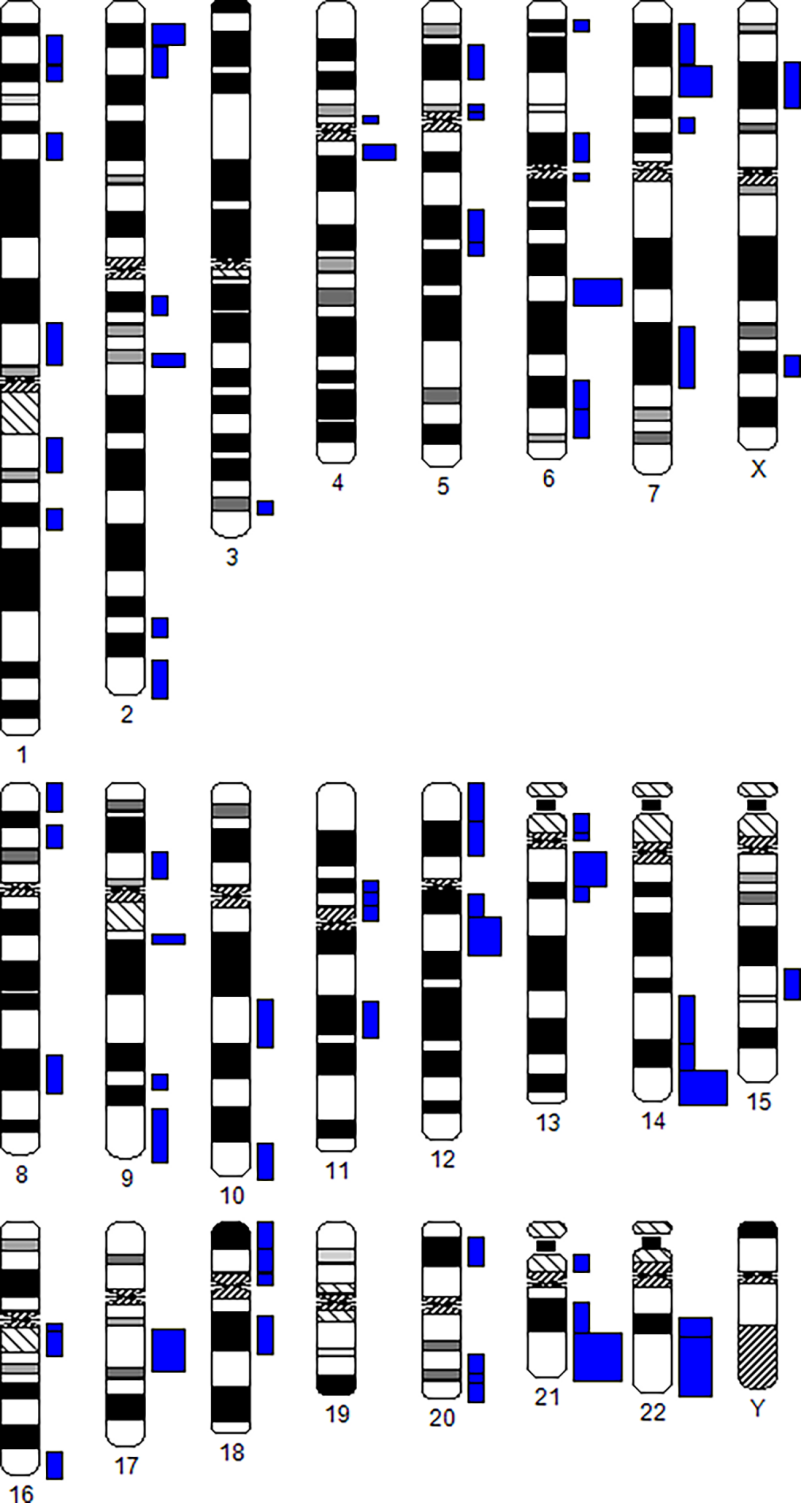
Localization of chromosomal translocations breakpoints in MSC. The width of the blue columns opposite the band corresponds to the number of breaks in this region.

Chromosomal aberrations were identified in 43 out of 406 analysed cells. Chromosome break points were registered in 93 cases. To calculate the number of chromosome break (chrb) events was considered as equal to two chromosome breaks (chtb) event as equal to one break, a deletion (del) event as equal to two breaks and a translocation (t) event as equal to two or more chromosome breaks. Localization of chromosome break points is shown in Fig 2. These breaks are probably random. Levels of chromosomal aberrations at different passages ranged from 6.7 to 16.1%, the average value was 10.13±1.27%, and the average level of chromosomal breaks per 1 cell was 0.21±0.04 (from 0.1 to 0.37). The number of chromosomal breaks was increased at passages 16 and 20, compared to the previous and subsequent passages (Table 2). There are no significant differences in the number of aberrant cells at different passages. This is due to the fact that there can be several lesions in one aberrant cell (e.g., translocation (t), deletion (del), see Table 1).

### Chromosome aberrations and clone formation

**Fig 3:**
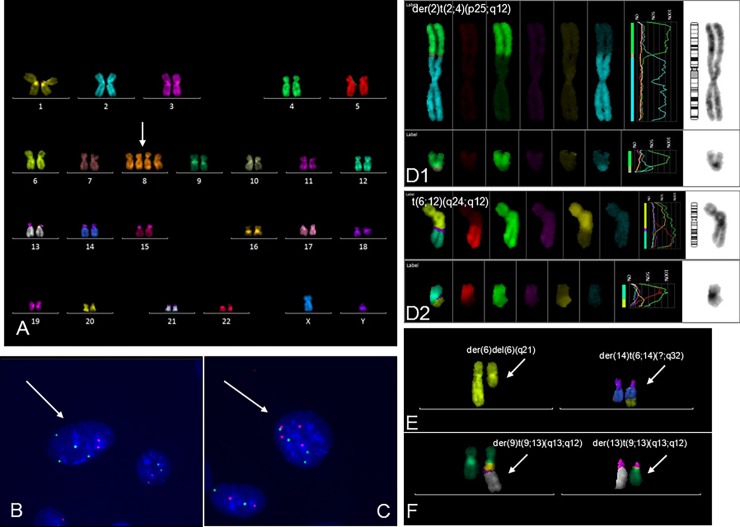
Clones with karyotypic abnormalities in MSC. A. Karyogram of the MSC clone with tetrasomy 8 (multicolor FISH, clone 1). B. The nucleus of MSC with 4 centromeric signals of chromosome 8 (green) and 2 chromosomes 6 (orange) (tetrasomy 8). C. The nucleus of MSC with 4 centromeric signals of chromosome 8 (green) and 4-chromosome 6 (orange) (polyploidy). Clonal chromosome translocations: D—clone 2 with two balanced translocations (D1 and D2), E—clone 3 with loss of part of chromosome 6, F—clone 4 with one balanced translocation.

Also, stable chromosomal translocations and random aberrations were observed (Table 1). According to the international cytogenetic nomenclature (ISCN-2013, section 11, "Neoplasia"), the clone must have at least two cells with the same chromosomal aberrations if the aberration is a chromosome gain or structural rearrangement, including initial and follow-up study (in our case, at different passages of cultivation). In case of a chromosomal loss, the same loss must be presented in at least three cells to be accepted as clonal. Thus, using the data of Table 1, three cell clones with structural chromosome aberrations in the examined MSC culture were identified. One of the clones 46,XY,t(2;4)(p24;q12),t(6;12)(q24;q12) contained two chromosomal reciprocal translocations between chromosomes (2;4) and (6;12). Cells from the clone were detected at passages 12, 18 and 20. Another clone 46,XY,del(6)(q21),t(6;14)(q?;q32) had an unbalanced translocation with partial deletions of the long arm of chromosome 6. The third clone 46,XY,t(9;13)(q13;q12) contained one reciprocal translocation between chromosomes 9 and 13. It was detected at passages 16 and 18 (Fig 3).

### Aneuploidy and clone formation

The same cells with aneuploidy (monosomy, trisomy) were found during the karyotype analysis using mFISH. Nine metaphases with Y chromosome monosomy, four with chromosome 9 monosomy, four with chromosome 22 monosomy, three with chromosome 12 monosomy and also nineteen with tetrasomy of chromosome 8 were detected at different passages. A clone with tetrasomy of chromosome 8 (Table 1) was identified at passages 16, 18 and 20 with frequencies 9.2, 15 and 3.3%, respectively. Because of the specific features of cytogenetic slides preparation, the evaluation of aneuploidy using interphase FISH is more preferable than the metaphase method. Interphase FISH was carried out only for the most numerous cell clones (with tetrasomy 8). Interphase FISH analysis was carried out using centromer-specific DNA probes for chromosomes 6 and 8 in order to confirm and determine the size of the clone with tetrasomy of chromosome 8. Moreover, the simultaneous evaluation of a pair of chromosomes allowed us to evaluate the effectiveness of hybridization and to perform an independent counting of diploid (ch6x2, ch8x2), aneuploid with tetrasomy 8 (ch6x2, ch8x4), and polyploid (ch6x4, ch8x4) cells. Using interphase FISH analysis, the aneuploid clone was already detected at passage 12 (0.8%), it reached the maximum value (12.6%) at passage 18 and decreased along with the reduction of proliferative activity by passage 26 (1.4%) (Table 3).

**Table 3:** A FISH-analysis with centromere-specific DNA probes of the chromosomes 6 and 8 in interphase nuclei.

passage	6x2 8x2	6x2 8x4	6x4 8x4	other variants	number of cells
6	98.6	0	1,2**	0.2	1366
12	96	0.8	3.1	0.1	1024
16	91.7	3.9*	4	0.4	1407
18	84.5	12.6*	2.6	0.3	1126
20	87.5	8.5*	3.6	0.4	1568
22	89.9	3.3*	6.2	0.6	1267
26	93.4	1.4	4.6	0.6	366

*—p <0.05 compared to the previous passage

**- p<0.05 compared to the passage 6

### Polyploidy

**Fig 4:**
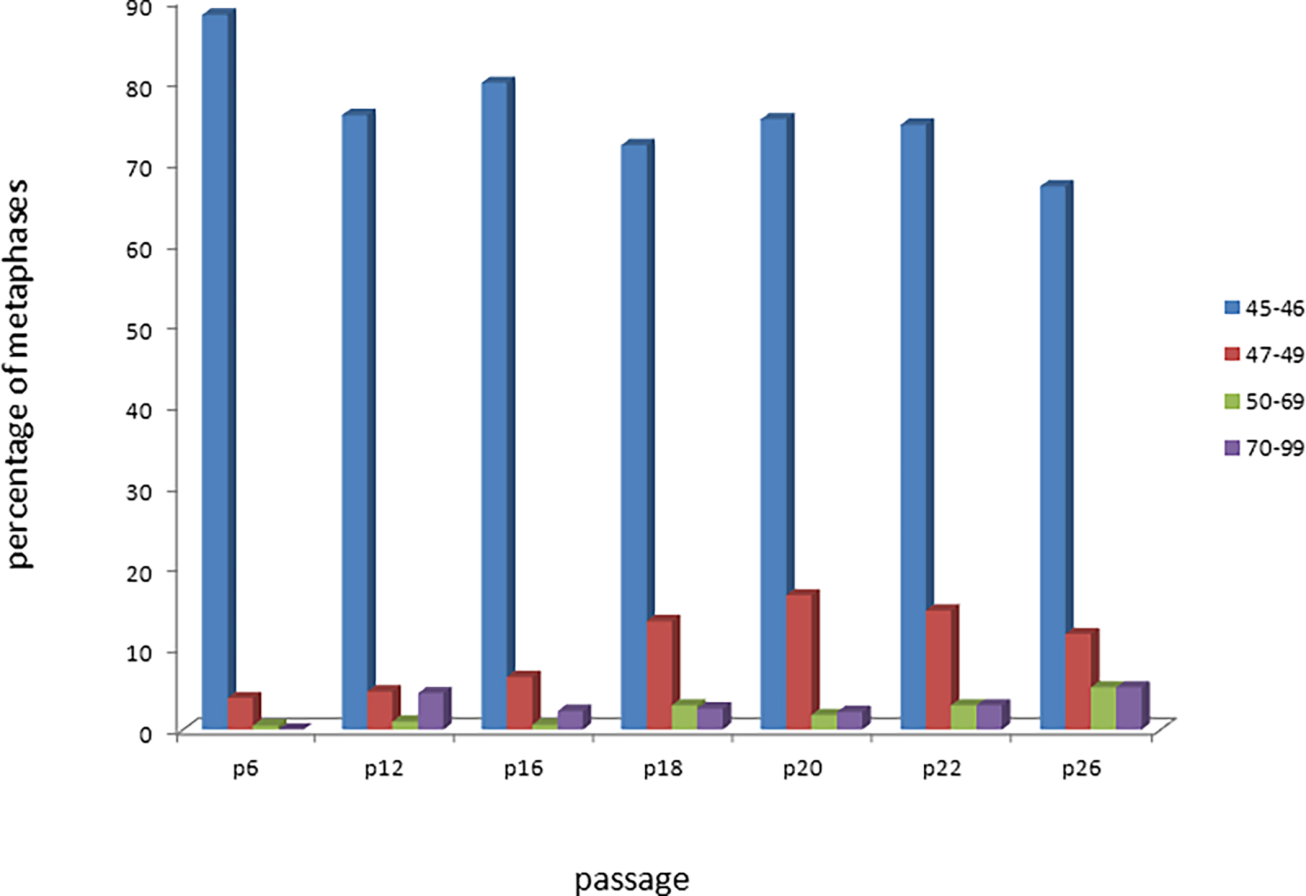
Histogram showing the distribution of chromosome counts in MSC at the different passages.

Polyploid cells were observed at all cultivation passages. The frequency of polyploid cells can be evaluated using a metaphase and interphase analysis. The level of metaphases with number of chromosomes from 50 to 99 was an average 5.73±1.05 (from 0.5% at passage 6 to 10.4% at passage 26) (Fig 4). Interphase FISH analysis was also carried out using centromere probes of chromosome 6 and 8. On average, 4.02 ±0.52% of polyploid cells was estimated starting from passage 12(Table 3). There is a small probability of random tetrasomy on two chromosomes at the same time, so if the nucleus had four signals of 6 and 8 chromosomes simultaneously, it was considered as a polyploidy event. Statistically significant difference in polyploidy frequency (p<0.05) was observed between passage 6 and all subsequent passages. The differences were confirmed through pairwise comparison.

## Discussion

A previously studied, genetically stable (up to passage 5) MSC culture, with a normal karyotype, was defrosted and cultivated until the mitotic activity significantly decreased (passage 26). Despite the significant differences in the number of chromosome breaks at some passages (Table 2) and although previous studies have indeed shown increased genomic instability in stem cell cultures that have been expanded for a prolonged period of time, the current study did not find a clear relationship between passage number and genomic instability. Breakpoints were heterogeneous from one batch to another (see Fig 2); they were probably random and characterized by the spontaneous level of chromosome aberrations in MSC. The level of spontaneous random chromosome aberrations estimated using mFISH was approximately 3 times higher than in previously studied MSC, obtained from adipose tissue analysed at early culture passages [3]. This can be attributed to both the duration of the cultivation and the difference in staining methods, since routine staining of chromosomes does not allow for the evaluation of the spectrum of chromosomal rearrangements as well as mFISH. The chromosome was counted according to the modal class principle (Fig 4), and the spread of values was discovered (45–99 chromosomes). Metaphases with more than 50 chromosomes are characterized by ploidy– 3n, 4n. The number of polyploid cells was 3.1% by passage 12 (Fig 4, Table 3) and stays almost invariable. In this study, polyploid cells were detected at all cultivation passages. The polyploidization (multiple sets of homologous chromosomes), including cells with clonal aberrations, is one of the most common ways of immortalized cell lines establishment. The rising amount of polyploid cells in the MSC line is an unfavourable sign for the cellular biomedical products.

**Fig 5:**
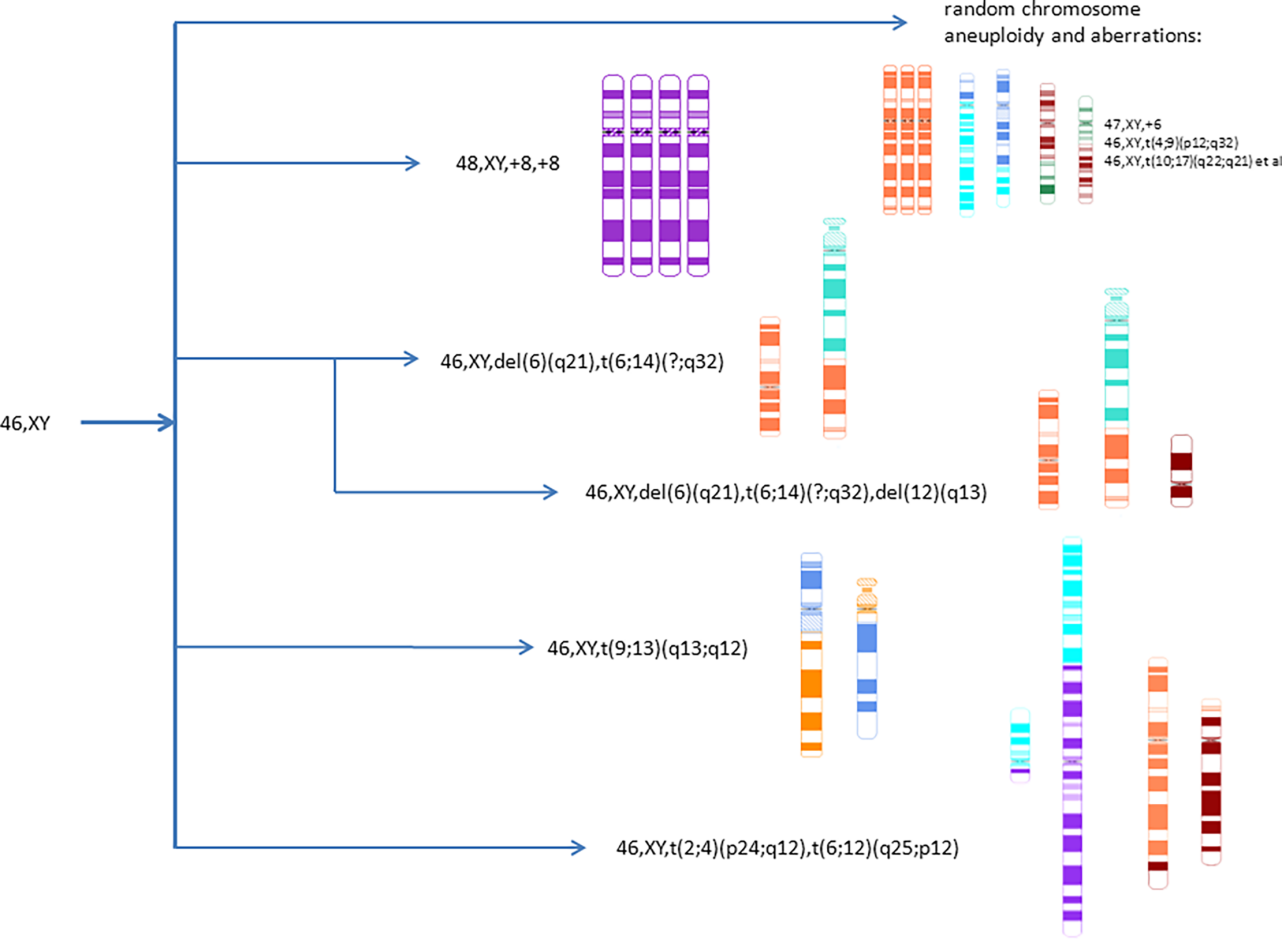
Schematic karyotype structure of MSC.

A clone of cells with tetrasomy 8 has been detected in this study. The number of cells with tetrasomy 8 was at its maximum (evaluated by centromere FISH probes) at passage 18. The cell clone with tetrasomy 8 was initially detected at passage 12, increased at passage 18 (12.6%) and was 1.4% at passage 26 (Table 3). Earlier, we have reported a similar clone with trisomy of chromosome 8 (trisomy 8) in one of bone marrow-derived MSC lines [12]. According to the literature, chromosome 8 polysomy (polysomy 8) is a common type of aneuploidy in human cells [19–22]. A high level of trisomy 8 occurs in extraembryonic tissues with confined placental mosaicism. This abnormality can be detected in 100% of newborns’ lymphocytes [19,20]. Besides, polysomy 8 is one of the frequent aberrations in malignant blood diseases; clones with trisomy 8 are resistant to chemotherapy and life expectancy of patients with trisomy 8 is lower than life expectancy of patients with normal karyotype [21,22]. An increase of chromosome 8 copy number leads to a dose increase of the c-MYC oncogene (located on the long arm of chromosome 8) and the enhancement of its expression, which apparently affects the proliferative potential of the cells. Moreover, it is shown that numerous differentiated human tissues are tolerant to the sufficiently high level of polysomies on chromosome 8 and cells with them are more stable than cells with aneuploidies on other chromosomes [19,20].The data on clonality in MSC suggest that processes of all cell lines formation *in vitro* are similar, and the degree of transformation is associated with the type of mutation. Unlike somatic cells *in vivo*, cell division and apoptotic errors are not regulated in cell lines *in vitro*. It leads to genetic instability and the formation of a large number of cells with different random chromosomal aberrations and, perhaps, new properties. Aberrant cells have to compete for habitat, and, clones and subclones of genetically different cells are forming. Chromosomal rearrangements that affect "driver" genes, for instance cell cycle checkpoints, oncogenes and tumour suppressors, can lead to the formation and progression of clonal subpopulations with significant selective advantage. Considering lessons learnt from establishment of continuous immortalized cell lines [1,2], it can be assumed that the clone selection proceeds through structural and epigenetic gene mutations, and structural chromosome aberrations are an instrument to achieve the gene balance, which is necessary for the existence of a cell population. Interestingly, it is chromosomal and genomic instability, and not point mutations that are typical for the majority of stable proliferating continuous cell lines. They have a certain karyotypic structure and can be characterized by a spectrum of rearranged marker chromosomes. In this study at least 4 cell clones with different chromosomal aberrations were identified and described (Table 1, Fig 5). Fig 5 schematically shows the structure of the MSC karyotype during prolonged cultivation. It should be noted that most of the cells have a normal karyotype, 46,XY, and only 1/6 part of cells are aberrant. However, clonal cells were also found among these aberrant cells. All of them differed in growth rate and, most probable, in their potential danger of spontaneous tumor transformation. The additional aberrations in the cell clones may indicate their genetic instability that is an unfavourable factor by analogy with the "clone progression" in oncohematological genetics. Thus, evaluation of the risks and benefits should be conducted before use of such cells in clinical practice. And to control the safety of cellular therapy, it is necessary not only to describe the constitutional karyotype, but also to estimate genetic stability and clone formation.
